# Antioxidant and Anti-Cancer Potentials of *Rheum emodi* Rhizome Extracts

**DOI:** 10.1093/ecam/neq048

**Published:** 2011-06-23

**Authors:** Venkatadri Rajkumar, Gunjan Guha, Rangasamy Ashok Kumar

**Affiliations:** Division of Biomedical Sciences, School of Bio Sciences and Technology, VIT University, Vellore 632014, India

## Abstract

The objective of this study was to determine antioxidant and cytotoxic efficacies of methanolic and aqueous extracts of *Rheum emodi* Wall. ex Meissn. rhizome. 2,2-Diphenyl-1-picrylhydrazyl (DPPH) and hydroxyl radical scavenging activities, inhibitory effect on lipid peroxidation and Fe^3+^ reducing antioxidant property have been used to investigate antioxidant properties of the extracts. Cytotoxicity of the extracts was tested on MDA-MB-435S and Hep3B cell lines. Both extracts displayed extensive cytotoxicity to the tested cell lines. The extracts were studied for their ability to protect pBR322 DNA from damage by UV induced photolysis of H_2_O_2_. The aqueous extract, though inferior to methanolic extract in its antioxidant potential exhibited efficiency in DNA protection, while the methanolic extract failed to protect the DNA. The amount of total polyphenolics in the extracts was measured by spectrophotometric method. The methanolic extract contained higher polyphenolic contents than aqueous extract. Significant positive correlations were observed (*P* < .05) between results of phenolic content estimation and that of antioxidant assays. Hence, high-performance liquid chromatography analysis was performed to identify few major phenolic compounds that might be responsible for these therapeutic properties. These results indicate that rhizome of *R. emodi* possesses antioxidant and cytotoxic activities and therefore have therapeutic potential.

## 1. Introduction

Cancer chemoprevention is defined as the use of natural, synthetic or biologic chemical agents to reverse, suppress, or prevent carcinogenic progression [1]. There have been increasing safety concerns over synthetic chemopreventive therapy. Commonly used synthetic antioxidants like butylated hydroxyanisole (BHA) and butylated hydroxyltoluene (BHT) have been restricted due to their toxicity and DNA damage induction potential [2, 3]. Instead, floral resources have received considerable attention as sources of biologically active substances including antioxidants, anti-mutagens and anti-carcinogens [4].

*Rheum emodi* Wall. ex Meissn. (Polygonaceae) is a leafy perennial herb distributed in altitudes ranging from 2800 to 3800 m in the temperate and subtropical regions of Himalayas from Kashmir to Sikkim in India [5]. Roots of *R. emodi* are reported to have antibacterial and antifungal activities [6–10]. In addition several other biological activities such as laxative, diuretic, and *in vivo* inhibitory effect towards P388 leukemia in mice are also reported [11–13].

The aim of this study was to determine the antioxidant and anti-cancer potential of methanolic and aqueous extracts of *R. emodi* rhizome by various methods including 2,2-diphenyl-1-picrylhydrazyl (DPPH) and ^•^OH radical scavenging, Fe^3+^ reducing capacity and inhibition of lipid peroxidation *in vitro* which are rarely reported. Cytotoxicity of the extracts were determined in MDA-MB-435S (human breast carcinoma) and Hep3B (liver carcinoma) cell lines. The protective activity of the extracts was evaluated by UV induced photolysis of H_2_O_2_ leading to damage to pBR322 DNA. Total phenolic content estimation was performed, and a few phenolic compounds that might be responsible for the antioxidant property of the extracts were identified by high-performance liquid chromatography (HPLC).

## 2. Methods

### 2.1. Chemicals and Other Reagents

DPPH, thiobarbituric acid (TBA), ethylene diamine tetraacetic acid (EDTA), ascorbic acid, trichloroacetic acid (TCA), phenazine methosulfate (PMS) (also known as *N*-methylphenazonium methosulfate), dimethyl sulfoxide (DMSO), L-15 (Leibovitz) cell culture medium (with l-glutamine), MEM (minimal essential medium) cell culture medium (with Earle's salt, NEAA and l-glutamine) and 2,4,6-tripyridyl-s-triazine (TPTZ) were purchased from Himedia Laboratories Pvt. Ltd. (India). XTT {2,3-*bis*(2-methoxy-4-nitro-5-sulfophenyl)-5-[(phenylamino) carbonyl]-2H-tetrazolium hydroxide} was obtained from Sigma Chemical Co. (St. Louis, MO, USA). Folin-Ciocalteau reagent was procured from Sisco Research Lab (India). The remaining chemicals and solvents used were of standard analytical grade and HPLC grade, respectively. pBR322 was purchased from Medox Biotech India Pvt. Ltd. (India). MDA-MB-435S (human breast carcinoma) and Hep3B (human liver carcinoma) cell lines were obtained from National Centre for Cell Science (Pune, India).

### 2.2. Plant Material

*Rheum emodi* rhizomes were collected from their natural habitat in the Garhwal Himalayas at Chamoli (30° 24′ N, 79° 21′ E), Uttaranchal, India in the month of June, 2007. Collected specimen were shade-dried, powdered and used for solvent extraction. Voucher specimen were maintained at our laboratory for future reference (Accession no.: VIT/SBCBE/CCL/07/6/04; Dated: June 11, 2007).

### 2.3. Extraction

Rhizome powder was serially extracted with methanol and water using a Soxhlet apparatus in a ratio of 1 : 6 [powder (in grams) : solvent (in milliliters)]. The extract obtained was evaporated to dryness at 40°C under reduced pressure (methanol: 337 mbar, aqueous: 72 mbar in a rotary evaporator (BÜchi, Switzerland). Fifty grams of rhizome powder yielded 24.81 g (percentage extract yield: 49.62% of dry weight) of crude methanolic extract and 3.86 g (percentage extract yield: 7.72% of dry weight) of crude aqueous extract. The samples were stored in a vacuum dessicator at room temperature until further use.

#### 2.3.1. DPPH Radical Scavenging Activity

Free radical scavenging ability of the extracts was tested by DPPH radical scavenging assay (DRSA) as described by Blois [14]. An amount of 20, 40, 60, 80 and 100 *μ*g of the extracts were taken in test tubes and made up to 0.5 ml with the respective solvents. An amount of 3 ml 0.1 mM DPPH^•^ in ethanol was added to each tube and incubated in dark at room temperature for 30 min. Absorbance was read at 517 nm using a Cary 50 UV-Vis Spectrophotometer (Varian Inc., Australia). Percentage DPPH radical scavenging activity (% DRSA) was calculated using the formula,

(1) $$\%\text{DRSA}=\left(\frac{A_{c}-A}{A_{c}}\right)\times 100,$$

where *A*_*c*_ is the absorbance of the control and *A* is the absorbance of the extract.

#### 2.3.2. Hydroxyl Radical Scavenging Activity

^•^OH radical scavenging activity (HRSA) of the extracts was estimated by the method of Klein et al. [15]. An amount of 50, 100, 150 and 200 *μ*g of the extracts were taken in test tubes. An amount of 1 ml iron-EDTA solution (0.13% ferrous ammonium sulfate and 0.26% EDTA), 0.5 ml of 0.018% EDTA and 1 ml of 0.85% (v/v) DMSO (in 0.1 M phosphate buffer, pH 7.4) were added followed by 0.5 ml of 0.22% (w/v) ascorbic acid. The tubes were capped tightly and incubated on a water bath at 85°C for 15 min. Post-incubation, the test tubes were uncapped and ice-cold trichloroacetic acid (17.5% w/v) was added in each immediately. An amount of 3 ml Nash reagent (7.5 g of ammonium acetate, 300 *μ*l glacial acetic acid and 200 *μ*l acetyl acetone were mixed and made up to 100 ml with distilled water) was added to all the tubes and incubated at room temperature for 15 min. Absorbance was measured at 412 nm.

Percentage hydroxyl radical scavenging activity (%HRSA) was calculated by the following formula:

(2) $$\%\text{HRSA}=\left(\frac{A_{c}-A}{A_{c}}\right)\times 100,$$

where *A*_*c*_ is the absorbance of the control and *A* is the absorbance of the extract.

#### 2.3.3. Ferric Reducing Antioxidant Property

Ferric reducing antioxidant property (FRAP) assay was done according to the protocol of Benzie and Strain [16] with some modifications. The stock solutions were 300 mM acetate buffer (with 16 ml C_2_H_4_O_2_; pH 3.6), TPTZ solution (10 mM TPTZ in 40 mM HCl) and 20 mM FeCl_3_·6H_2_O solution. Working FRAP solution was prepared freshly by mixing 25 ml of acetate buffer, 2.5 ml TPTZ solution and 2.5 ml of FeCl_3_·6H_2_O solution, and then warmed to 37°C before use. An amount of 150 *μ*l individual extract solutions (containing 25, 50, 100 and 200 *μ*g of extracts, resp.) was allowed to react with 2.85 ml of FRAP solution for 30 min in dark. Absorbance was read at 593 nm. Percentage Fe^3+^ reduction (to Fe^2+^) was calculated by a FeSO_4_ standard calibration curve. Percentage scavenging was also evaluated in ascorbic acid equivalence (AAE) (in micrograms).

#### 2.3.4. Thiobarbituric Acid Assay

The assay was performed as described by Halliwell and Gutteridge [17], in which the extent of lipid peroxidation was estimated from the concentration of malondialdehyde (MDA), a thiobarbituric acid reactive substance (TBARS), which is produced due to lipid peroxidation. A 6-week-old female Wistar albino rat weighing *∼*150 g was sacrificed under ethereal anesthesia and its liver was excised. 10% (w/v) liver homogenate was prepared in Dulbecco's phosphate buffered saline (PBS) (Ca^2+^/Mg^2+^-free) (pH 7.4), and centrifuged at 503 g for 15 min to obtain a clear supernatant. An amount of 50, 100, 150, 200 and 250 *μ*g of the extracts were taken in test tubes and were evaporated to dryness at 80°C. An amount of 1 ml 0.15 M potassium chloride was added to the tubes followed by 0.5 ml of rat liver homogenate (10% w/v in PBS). Peroxidation was initiated by the addition of 100 *μ*l of 2 mM ferric chloride. After incubating the tubes for 30 min at 37°C, the peroxidation reaction was stopped by adding 2 ml of ice-cold HCl (0.25 N) containing 15% TCA and 0.38% TBA. The tubes were kept at 80°C for 1 h, cooled and centrifuged at 3144 g. The absorbance of the supernatant, containing TBA-MDA complex was read at 532 nm. Percentage inhibition of lipid peroxidation (%LPI) was calculated using the formula,

(3) $$\%\text{LPI}=\left(\frac{A_{c}-A}{A_{c}}\right)\times 100,$$

where *A*_*c*_ is the absorbance of the control and *A* is the absorbance of the extract.

This experiment was performed according to the guidelines of the “European Convention for the Protection of Vertebrate Animals used for Experimental and Other Scientific Purposes" (and its appendix) with the approval of the institutional animal ethical committee (PSGIMSR/27.02.2008).

#### 2.3.5. XTT Assay

XTT assay was performed on MDA-MB-435S (grown in L-15 medium) and Hep3B (grown in MEM medium) cell lines as described by Weislow et al. [18]. A total of 6 × 10^3^ cells were seeded on 96-well plates and were supplemented with 200 *μ*l of the respective culture media for a period of 24 h. The media were then substituted by 200 *μ*l of fresh media containing varying concentrations of the extracts (15.625, 31.25, 62.5 and 125 *μ*g/ml). The plates were incubated at 37°C for 24 h, after which, media was removed and fresh media added. An amount of 50 *μ*l XTT reagent prepared in respective media (0.6 mg/ml) containing 25 *μ*M of PMS was then added to all the wells, and the plates were incubated in dark humid conditions at 37°C for 4 h. After incubation, the orange colored complex formed was read at 450 nm using a Dynex Opsys MR Microplate Reader (Dynex Technologies, VA, USA) with a 630 nm reference filter. Wells containing cells without extract treatments served as the control. Wells containing only culture medium and XTT reagent served as the blank. Percentage cytotoxicity of the extracts was calculated by using the formula:

(4) $$\%\text{Cytotoxicity}=\left(\frac{A_{c}-A}{A_{c}}\right)\times 100,$$

where *A*_*c*_ is the absorbance of the control and *A* is the absorbance of the sample.

### 2.4. DNA Damage Inhibition Efficiency

Potential DNA damage inhibition by *R. emodi* extracts was tested by photolysing H_2_O_2_ by UV radiation in presence of pBR322 plasmid DNA and performing agarose gel electrophoresis with the irradiated DNA [19]. A total of 1 *μ*l aliquots of pBR322 (200 *μ*g/ml) were taken in UV non-resistant polyethylene microcentrifuge tubes. A quantity of 50 *μ*g of each extract was separately added to two tubes. The remaining tube was left untreated as the irradiated control (C_R_). An amount of 4 *μ*l of 3% H_2_O_2_ was added to all the tubes which were then placed directly on the surface of a UV transilluminator (300 nm). The samples were irradiated for 10 min at room temperature. After irradiation, 4 *μ*l of tracking dye (0.25% bromophenol blue, 0.25% xylene cyanol FF and 30% glycerol) was added. The samples in all tubes were then analyzed by gel electrophoresis on a 1% agarose gel in TBE buffer (pH 8). Untreated non-irradiated pBR322 plasmid (C) was run along with the extract-treated UV-irradiated samples (methanolic extract treated = S_M_ and aqueous extract treated = S_A_) and untreated UV-irradiated (C_R_) plasmid DNA. The gel was stained in ethidium bromide (1 *μ*g/ml; 30 min) and photographed on Lourmat Gel Imaging System (Vilbar, France).

#### 2.4.1. Estimation of Total Phenolic Content

Total phenolic content was determined by the method described by Lister and Wilson [20]. An amount of 50, 100, 150, 200 and 250 of the extracts were made up to 0.5 ml with distilled water. An amount of 2.5 ml Folin-Ciocalteau reagent (1 : 10 dilution) and 2 ml of sodium carbonate (7.5% w/v) were added and the tubes incubated at 45°C for 15 min. Absorbance were read at 765 nm using a Cary 50 UV-Vis spectrophotometer (Varian, Inc., CA, USA). Results were expressed in terms of gallic acid equivalence (GAE) in micrograms.

#### 2.4.2. HPLC Analysis for Phenolic Compounds

HPLC analysis was performed using a Waters 2487 HPLC system consisting of a dual *λ* detector and a Waters 1525 binary pump, and equipped with a Waters Symmetry C18 column (5 *μ*m, 4.6 × 150 mm) with Waters Sentry universal guard column (5 *μ*m, 4.6 × 20 mm) (Waters Corporation, Milford, MA, USA). Phenolic compounds in the aqueous and methanolic extracts of *R. emodi* were analyzed using the library for phenolic compound standards [21] as a reference. Gradient elution was performed at 35°C with Solution A (50 mM sodium phosphate in 10% methanol; pH 3.3) and Solution B (70% methanol) in the following gradient elution program: 0–15 min—100% of Solution A; 15–45 min—70% of Solution A; 45–65 min—65% of Solution A; 65–70 min—60% of Solution A; 70–95 min—50% of Solution A; 95–100 min—0% of Solution A. Flow rate was 1 ml/min and injection volume was 20 *μ*l. Detection was monitored at diverse wavelengths (around *λ*_max_) for various phenolic compounds, that is, 250 nm for benzoic acids, isoflavones and most anthraquinones; 280 nm for some flavones, flavanones, catechins, theaflavins and some anthraquinones; 320 nm for cinnamic acids, most flavones and chalcones; 370 nm for flavonols; 510 nm for anthocyanins.

### 2.5. Statistical Analysis

All analyses were carried out in triplicates. Data were presented as mean ± SD. Statistical analyses were performed by one-way ANOVA. Significant differences between groups were determined at *P* < .05. To evaluate relationships between experimental parameters, results were analyzed for correlation and regression and tested for significance by Student's *t*-test (*P* < .05). MATLAB ver. 7.0 (Natick, MA, USA), GraphPad Prism 5.0 (San Diego, CA, USA) and Microsoft Excel 2007 (Roselle, IL, USA) were used for the statistical and graphical evaluations.

## 3. Results

### 3.1. DRSA

**Figure 1:**
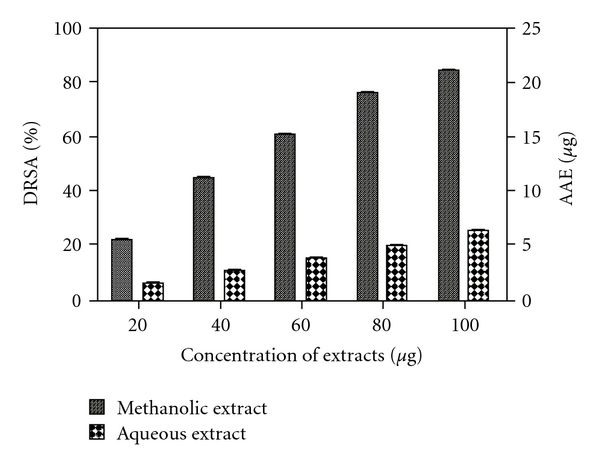
%DRSA of methanolic and aqueous extracts of *R. emodi* rhizome with AAE in micrograms. Data expressed as mean ± SD of *n* = 3 samples (*P* < .05) for all tested dosages.

Both extracts showed a concentration dependent scavenging of DPPH^•^ radicals. Methanolic extract was found to be more active radical scavenger than aqueous extract. Results were plotted as %DRSA and also expressed as AAE in micrograms (Figure 1).

### 3.2. HRSA

**Figure 2:**
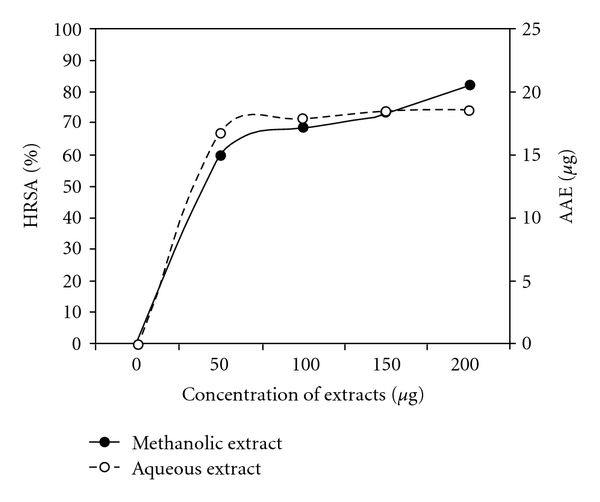
Mean %HRSA of methanolic and aqueous extracts of *R. emodi* rhizome (*P* < .05).

The ability of the extracts to quench ^•^OH radicals can be related to the prevention of lipid peroxidation, and it seems to be a good scavenger of active oxygen species, thus reducing the rate of chain reaction.  Figure 2 shows %HRSA of the two extracts. Hydroxyl radical has been implicated as highly damaging in free-radical pathology, capable of damaging almost every molecule found in living cells [22]. The extracts have shown a dosage-dependent increase in inhibition of ^•^OH radicals.

### 3.3. FRAP

**Figure 3:**
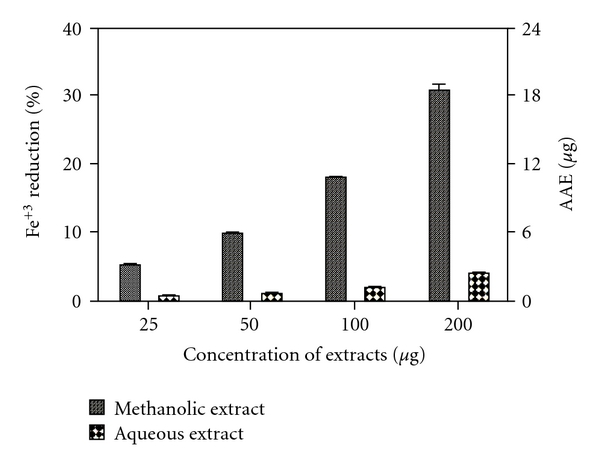
Percentage Fe^3+^ reduction by methanolic and aqueous extracts of *R. emodi* rhizome with AAE in micrograms. Data expressed as mean ± SD (*n* = 3, *P* < .05) for all tested dosages.

Fe^3+^ reducing activity of the two extracts were determined by FRAP assay. The methanolic extract showed higher reducing power in comparison to the aqueous extract for all tested dosages.  Figure 3 shows %Fe^3+^ reduction by both extracts along with AAE in micrograms.

### 3.4. TBA Assay

**Figure 4:**
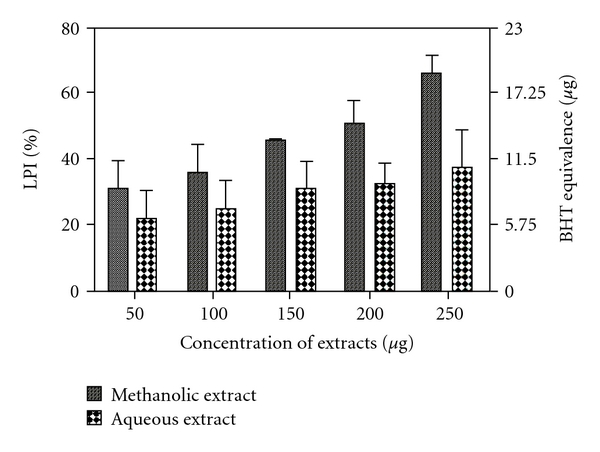
%LPI by methanolic and aqueous extracts of *R. emodi* rhizome with BHT equivalence in micrograms. Data expressed as mean ± SD (*n* = 3, *P* < .05) for all dosages.

Both extracts were capable of preventing formation of MDA in a dosage-dependent manner. The methanolic extract was observed to be a significantly better inhibitor of lipid peroxidation (at *P* < .05) compared with the aqueous extract for all tested dosages.  Figure 4 shows %LPI with their corresponding BHT equivalences (in micrograms).

**Figure 5:**
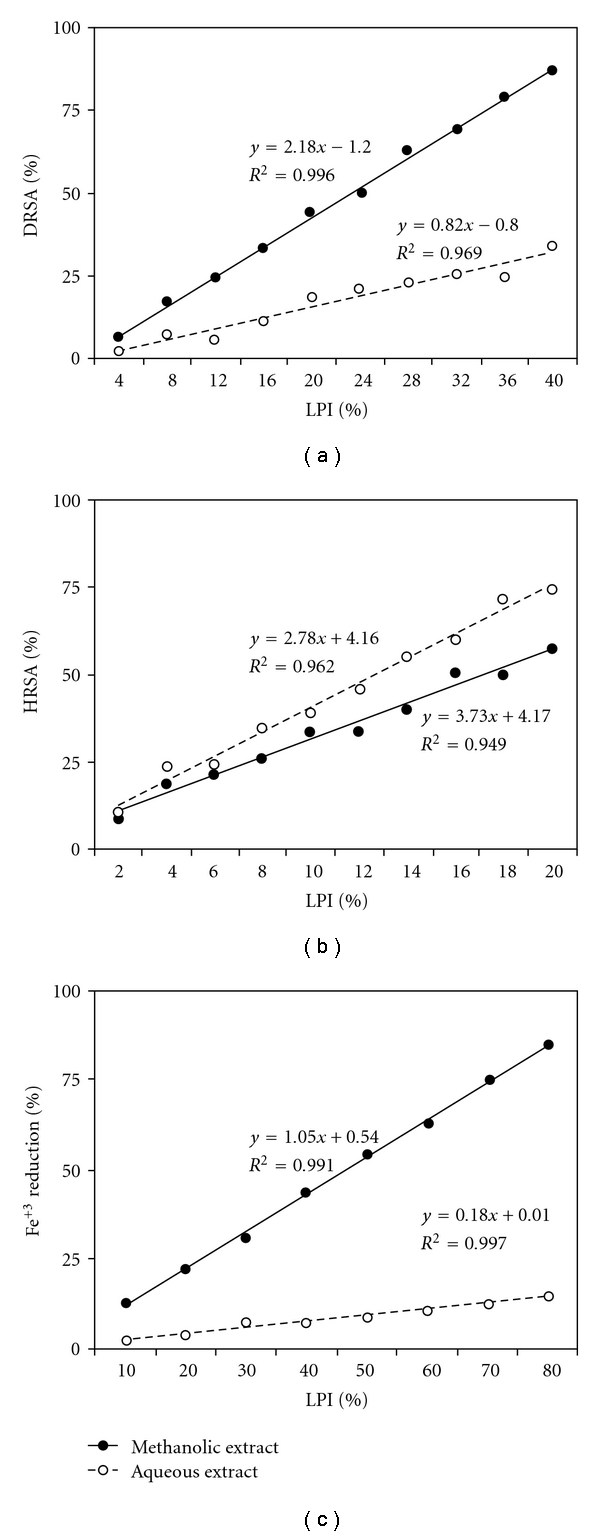
Methanolic and aqueous extracts of *R. emodi* rhizome inhibit lipid peroxidation by virtue of their radical scavenging potential. Significant correlations (*P* < .05) observed between %LPI and (a) %DRSA, (b) %HRSA, (c) percentage Fe^3+^ reducing potential.

Significant correlations (*P* < .05) were observed between the following: (i) %LPI and %DRSA, (ii) %LPI and %HRSA and (iii) %LPI and %Fe^3+^ reductions (Figure 5) for both methanolic and aqueous extracts for all dosages. This infers that both extracts differentially inhibit lipid peroxidation by virtue of their varying degrees of free radical quenching potential.

### 3.5. XTT Assay

Both the extracts demonstrated considerable cytotoxicity in both cell lines, thereby indicating the presence of anti-cancer metabolites.  Table 1 presents the IC_50_ values for the aqueous and methanolic extracts of *R. emodi* in MDA-MB-435S and Hep3B cell lines.

### 3.6. DNA Damage Inhibition Efficiency

**Figure 6:**
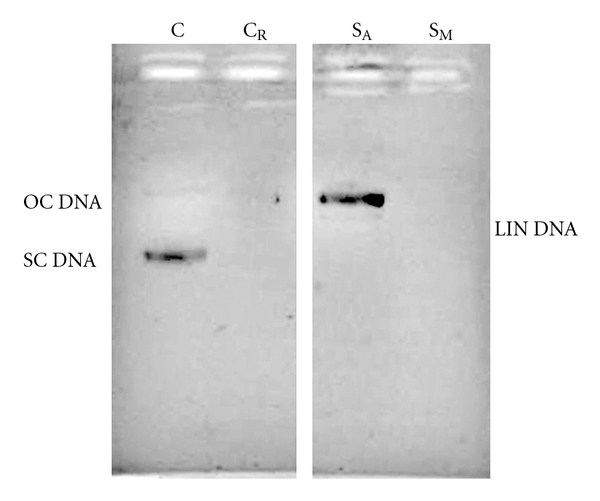
Effect of *R. emodi* rhizome extracts (50 *μ*g) on the protection of supercoiled DNA (pBR322) against oxidative damage caused by UV-photolysed H_2_O_2_ (3%). C = untreated non-irradiated DNA (control); C_R_ = untreated UV-irradiated DNA (control); S_A_ = UV-irradiated, aqueous extract treated; S_M_ = UV-irradiated, methanolic extract treated; SC DNA = supercoiled DNA; OC DNA = open circular DNA; LIN DNA = linear DNA.

Figure 6 shows the electrophoretic pattern of pBR322 DNA following UV-photolysis of H_2_O_2_ in absence (in controls C and C_R_) and presence (in samples S_A_ and S_M_) of the extracts. Control pBR322 (C) showed two bands on agarose gel electrophoresis. The faster moving band represented the native form of supercoiled circular DNA (SC DNA) and the slower moving faint band corresponded to the open circular form (OC DNA) [19]. The aqueous extract displayed considerably protective activity in comparison to the methanolic extract which did not show any protective activity. UV-photolysis of H_2_O_2_ in C_R_ damaged the entire DNA (no bands visible). S_A_ did not show a SC DNA band; instead, it developed a new faint intermediate band for linear DNA (LIN DNA) as a result of free radical damage to SC DNA. On the other hand, the methanolic extract did not show any protective activity. The results infer that UV-photolysed H_2_O_2_ (3%) treatment of pBR322 obliterated the entire DNA (in C_R_), while 50 *μ*g of the aqueous extract gave partial protection against DNA damage.

It is, however, an interesting observation that albeit identical ^•^OH radical scavenging potential of both extracts, the methanolic extract failed comprehensively to encounter the effects of UV-photolysed H_2_O_2_-derived ^•^OH radicals that cause oxidative DNA damage. This letdown might be due to a probable DNA-damaging property of the methanolic extract per se. Cytotoxicity of the methanolic extract, as already observed in this study, is about three to four times more than that of aqueous extract (Table 1). Such extensive cytotoxicity of the methanolic extract might be hypothesized to be a probable function of its DNA-damaging capacity.

#### 3.6.1. Estimation of Total Phenolic Content

**Figure 7:**
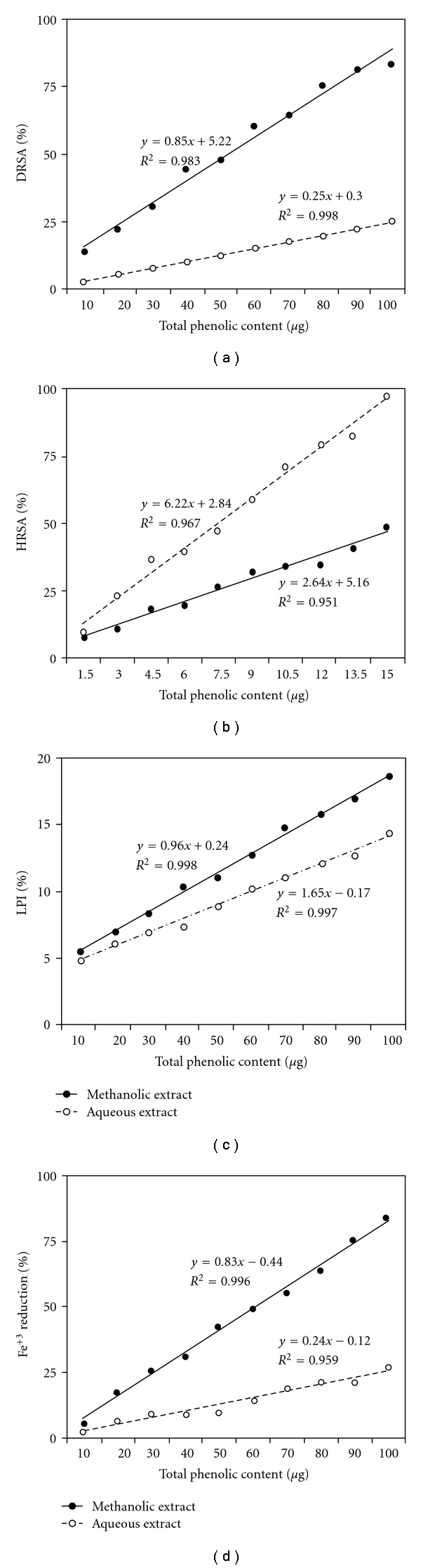
Relationship between total phenolic content of methanolic and aqueous extracts of *R. emodi* rhizome and their (a) %DRSA, (b) %HRSA, (c) %LPI, (d) percentage Fe^3+^ reducing potential. All parameters show strong and significant positive correlation with total phenolic content (at *P* < .05) for both extracts.

Table 2 shows the total phenolic contents in the methanolic and aqueous extracts expressed as GAE (in micrograms). The results obtained in all antioxidant assays showed statistically significant difference between the methanolic and aqueous extracts at *P* < .05. Correlation and regression analyses were performed to check whether the polyphenols in the extracts are responsible for these activities. Total phenolic content of both extracts showed significant and strong positive correlation (*P* < .05) with free radical (DPPH^•^ and ^•^OH) scavenging efficiencies, %LPI and %Fe^3+^ reductions (Figure 7). These results suggest a probable paramount role that the polyphenolic constituents of the extracts might play in free radical neutralization and lipid peroxidation inhibition.

#### 3.6.2. HPLC Analysis

Due to the diversity and complexity of natural phenolic compounds, it is difficult to characterize every compound present in the crude extract to elucidate its structure [23]. Major types of phenolic compounds were determined in the two extracts of *R. emodi* by HPLC analysis. A library of the analytical characteristics (*λ*_max_, retention time, determining *λ*, slope and limit calibration) of more than 100 phenolic standards established by Sakakibara et al. [21] was used as a reference for compound identification.  Table 3 shows the phenolic compounds identified in the methanolic extract of *R. emodi* rhizome along with the respective retention times (*R_t_*). Both aqueous and methanolic extracts also contained unknown compounds evident from the HPLC data whose characterization is in prospect. 

## 4. Discussion

Aqueous and methanolic extracts of *R. emodi* showed promising antioxidant activity in all the experimental models used. Both extracts were found to have a dosage-dependent increase in their antioxidant potentials with varying degrees of efficiencies. The differential scavenging activities of the extracts may be attributed to the varying mechanisms of radical scavenging in these assays. The extracts were observed to be good scavengers of hydroxyl radical, which is involved in DNA crosslinkings and strand breaks, and is considered to be one of the quick initiators of lipid peroxidation [24]. The ability of the extracts to quench hydroxyl radicals might directly relate to the prevention of lipid peroxidation. It can be inferred that the extracts might prevent reactive radical species from damaging biomolecules such as lipoproteins, polyunsaturated fatty acids, DNA, amino acids, proteins and sugars in biological systems [25].

Both extracts showed concentration-dependent cytotoxicity when tested in each of the two cancer cell lines. According to the American National Cancer Institute, the IC_50_ value to consider a crude extract promising for development of anti-cancer drug(s) is lower than a limit threshold of 30 *μ*g/ml [26]. The extracts can thus serve as potential source for anti-cancer compounds. The aqueous extract, on the other hand, although has lower potential as a cytotoxin, shows considerable degree of DNA protection against oxidative damage, while its methanolic counterpart holistically lacks this property. These differences can be attributed to the presence of differential protective metabolites eluted out in the two solvents, and also due to factors like stereoselectivity and/or solubility of the two extracts [27]. Both extracts were therefore found to have promising potential towards the development of drugs that might be used to target tumors for chemoprevention/chemotherapy to check neoplastic growth and malignancy.

A significant (*P* < .05) positive correlation was extrapolated between the results of the assay for estimation of total phenolic content and those investigating other therapeutic parameters. In view of this, HPLC analysis was performed to identify some of the major phenolic compounds in both extracts. However, we accomplished in identifying major polyphenols only in the methanolic extract.

**Figure 8:**
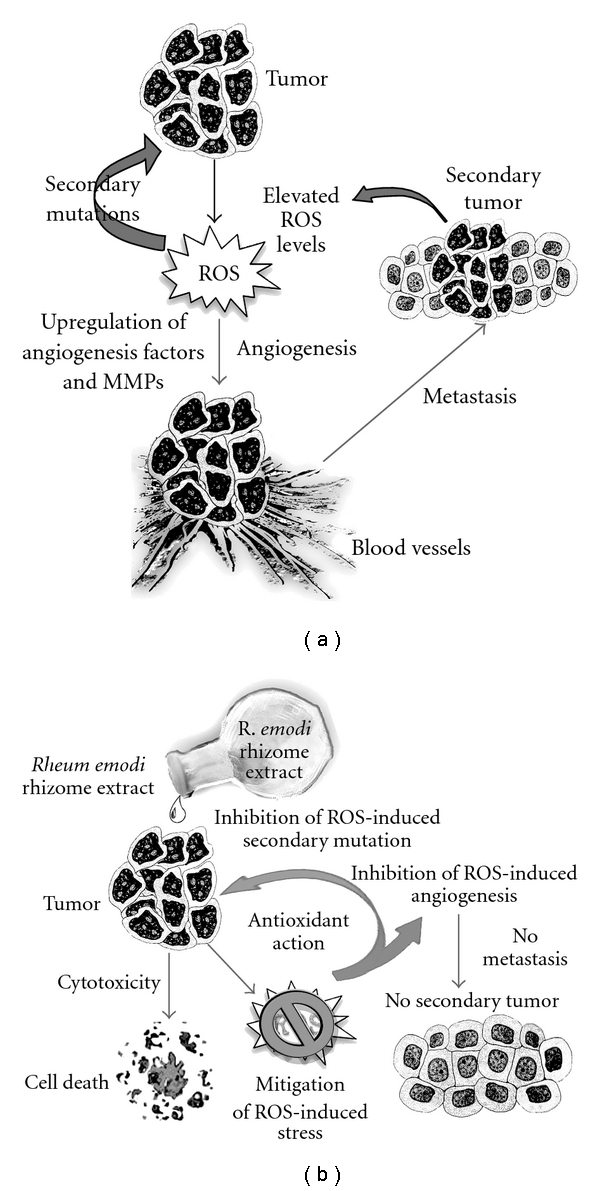
Hypothetical diagram representing the modes of action of *R. emodi* rhizome extracts as an anti-cancer agent. (a) Progression of cancer through ROS generation, and consequent secondary mutations, angiogenesis and metastasis. (b) *Rheum emodi* rhizome extracts prevents cancer progression through cytotoxicity and antioxidant potential.

The antioxidant, cytotoxicity and DNA protection abilities of the extracts render them suitable to be considered as a source for the development of anti-cancer drugs (Figure 8). In tumor cells, ROS is produced extensively, which thereby increases levels of certain growth factors and enzymes like metalloproteinases (MMPs) which promote angiogenesis, and also elevates the risk of metastasis and development of secondary tumors [28]. Antioxidant properties of the extracts might therefore prevent progression of cancer; while the cytotoxic potential, on the other hand, might be used against cancer cells, thereby directing them towards apoptosis and cell death. DNA protection property might hold good in inhibiting secondary mutations in progressive tumor tissues.

## 5. Conclusion

Rhizome of *R. emodi* might be a potential source for anti-cancer metabolites which can be mustered for the development of effective cancer drugs. Isolation and characterization of compounds from *R. emodi* rhizome extracts are in prospect.

## Figures and Tables

**Table 1:** IC_50_ values of aqueous and methanolic extracts of *R. emodi* in MDA-MB-435S and Hep3B cell lines as determined in XTT assay.

	MDA-MB-435S (*μ*g ml^−1^)	Hep3B (*μ*g ml^−1^)
Aqueous extract	33.00 ± 8.31	85.58 ± 3.60
Methanolic extract	8.50 ± 3.70	38.43 ± 6.00

**Table 2:** Total phenolic contents of methanolic and aqueous extract of *R. emodi* rhizome.

Concentration (*μ*g)	GAE ± SD (*μ*g)^(a)^	
	Methanolic extract	Aqueous extract
50	13.33 ± 0.18	7.68 ± 0.073
100	20.96 ± 0.23	9.94 ± 0.016
150	28.98 ± 0.38	12.13 ± 0.092
200	36.44 ± 0.26	14.30 ± 0.016
250	36.99 ± 0.53	14.71 ± 0.028

^(a)^GAE ± SD at 95% confidence interval.

**Table 3:** Major phenolic compounds identified in the methanolic extract of *R. emodi* rhizome by HPLC.

Phenolic compounds	*λ* ^(a)^ (nm)	ET_R_^(b)^ (min)	RT_R_^(c)^ (min)
*β*-Resorcylic acid	250	10.896	10.9
Daidzein-8-*O*-glucoside (puerarin)	250	19.703	20.1
Daidzein	250	63.828	64.1
(+)-Taxifolin	280	26.696	26.7
Quercetin	370	75.008	75.5
Flavonol	370	91.337	91.5

^(a)^Wavelenghth for determination.

^(b)^Analysis retention times.

^(c)^Reference retention times [21].
